# Alpha-power in electroencephalography as good outcome predictor for out-of-hospital cardiac arrest survivors

**DOI:** 10.1038/s41598-022-15144-3

**Published:** 2022-06-28

**Authors:** Min-Jee Kim, Youn-Jung Kim, Mi-Sun Yum, Won Young Kim

**Affiliations:** 1 Division of Pediatric Neurology, Department of Pediatrics, Asan Medical Center Children’s Hospital, Ulsan University College of Medicine, 88, Olympic-ro 43-gil, Songpa-gu, Seoul, 05505 Korea; 2 Department of Emergency Medicine, Asan Medical Center, Ulsan University College of Medicine, 88, Olympic-ro 43-gil, Songpa-gu, Seoul, 05505 Korea

**Keywords:** Computational biology and bioinformatics, Neuroscience, Biomarkers, Medical research, Neurology

## Abstract

This study aimed to investigate the utility of quantitative EEG biomarkers for predicting good neurologic outcomes in OHCA survivors treated with targeted temperature management (TTM) using power spectral density (PSD), event-related spectral perturbation (ERSP), and spectral entropy (SE). This observational registry-based study was conducted at a tertiary care hospital in Korea using data of adult nontraumatic comatose OHCA survivors who underwent standard EEG and treated with TTM between 2010 and 2018. Good neurological outcome at 1 month (Cerebral Performance Category scores 1 and 2) was the primary outcome. The linear mixed model analysis was performed for PSD, ESRP, and SE values of all and each frequency band. Thirteen of the 54 comatose OHCA survivors with TTM and EEG were excluded due to poor EEG quality or periodic/rhythmic pattern, and EEG data of 41 patients were used for analysis. The median time to EEG was 21 h, and the rate of the good neurologic outcome at 1 month was 52.5%. The good neurologic outcome group was significantly younger and showed higher PSD and ERSP and lower SE features for each frequency than the poor outcome group. After age adjustment, only the alpha-PSD was significantly higher in the good neurologic outcome group (1.13 ± 1.11 vs. 0.09 ± 0.09, *p* = 0.031) and had best performance with 0.903 of the area under the curve for predicting good neurologic outcome. Alpha-PSD best predicts good neurologic outcome in OHCA survivors and is an early biomarker for prognostication. Larger studies are needed to conclusively confirm these findings.

## Introduction

More than 250,000 people in the USA and Europe annually experience out-of-hospital cardiac arrest (OHCA)^1,2^ with only 8.3% of survivors having a favorable neurological outcome and being eligible for discharge^2^. Hypoxic-ischemic brain injury after the decision to withdraw life-sustaining therapy (WLST) is the leading cause of mortality, and accurate prediction of neurological outcomes is of utmost importance to guide the nature and duration of therapeutic interventions^3,4^. Recent recommendations proposed the strategy for predicting the neurological outcome based on a multimodal approach using clinical examination, electrophysiology, biomarkers, and brain imaging^5–7^.

Electroencephalography (EEG) is the most widely used and easily accessible tool to predict prognosis and detect subclinical seizures after cardiac arrest^8^. Although the standardized critical care EEG terminology proposed by American Clinical Neurophysiology Society (ACNS) and the standard EEG classification (highly malignant, malignant, and benign pattern), its clinical usability is limited by poor reproducibility and low inter-rater reliability even among experts^9–16^. Some specific EEG patterns or features were suggested for prognostication after cardiac arrest^17,18^, and various confounders including examination timing, the patient’s condition, and different sedatives or anesthetics used in these patients interfere with the objective identification of these features by visual inspection^18,19^. These limitations encourage the development of quantitative EEG biomarkers for neurologic outcome prediction in OHCA instead of expert interpretation.

To quantify the background EEG with standard recording, we analyzed the power, variability and randomness of each frequency band using the power spectral density (PSD), event-related spectral perturbation (ERSP), and spectral entropy (SE) in patients with OHCA treated with targeted temperature management (TTM). Finally, readily available and accurate quantitative biomarker for good outcomes was proposed with optimal cutoff values.

## Results

Among 54 nontraumatic OHCA survivors who received TTM and underwent EEG, two and 11 patients were excluded due to poor ECG quality, periodic or rhythmic pattern, respectively. Thus, 41 patients were ultimately included in this study. Patients were categorized into the good (*n* = 21, 52.5%) and poor (*n* = 20, 47.5%) neurological outcome groups, respectively.

### Demographic and clinical characteristics of patients

The demographic and clinical characteristics of the patients are summarized in Table 1. The patients in the good neurological outcome group were younger (mean, 51.9 vs. 65.2 years; *P* = 0.012) and more frequently male (85.7% vs. 50.0%; *P* = 0.014). The patients in the good neurological outcome group also showed higher frequency of witnessed arrest (85.6% vs. 50.0%; *P* = 0.014), initial shockable rhythm (85.7% vs. 30.0%; *P* < 0.001), and shorter resuscitation duration (median, 11.0 vs. 34.0 min; *P* = 0.022).

**Table 1:** Comparison of the clinical characteristics between the out-of-hospital cardiac arrest patients with good and poor neurologic outcomes at 1 month.

Characteristics	Total (*n* = 41)	Good neurologic outcome (*n* = 21)	Poor neurologic outcome (*n* = 20)	*p* value
Age, years	58.4 (17.3)	51.9 (14.9)	65.2 (17.3)	0.012
Male	28 (68.3%)	18 (85.7%)	10 (50.0%)	0.014
**Previous medical history**				
No comorbid disease	13 (31.7%)	10 (47.6%)	3 (15.0%)	0.025
Hypertension	10 (24.4%)	4 (19.0%)	6 (30.0%)	0.484
Diabetes mellitus	7 (17.1%)	2 (9.5%)	5 (25.0%)	0.238
Congestive heart failure	3 (7.3%)	1 (4.8%)	2 (10.0%)	0.606
Chronic kidney disease	3 (7.3%)	0 (0%)	3 (15.0%)	0.107
**Arrest characteristics**				
Presence of a witness	28 (68.3%)	18 (85.7%)	10 (50.0%)	0.014
Bystander CPR	25 (61.0%)	14 (66.7%)	11 (55.0%)	0.444
Initial shockable rhythm	24 (58.5%)	18 (85.7%)	6 (30.0%)	< 0.001
No flow time, min	0.0 (0.0–5.0)	0.0 (0.0–8.5)	1.0 (0.0–4.5)	0.877
Resuscitation duration, min	18.0 (7.5–37.5)	11.0 (6.5–24.5)	34.0 (11.0–41.5)	0.022
Time from ROSC to target temperature, min	437 (236.4)	423 (189.5)	453 (284.0)	0.696
Time from ROSC to EEG, hours	21.0 (11.5–37.0)	19.0 (8.5–39.5)	22.5 (17.3–36.8)	0.481
Treated sedative	41 (100.0%)	21 (100.0%)	20 (100.0%)	1.000
Propofol	36 (87.8%)	19 (90.5%)	17 (85.0%)	0.486
Midazolam	6 (14.6%)	5 (23.8%)	1 (5.0%)	0.102
Fentanyl	37 (90.2%)	19 (90.5%)	18 (90.0%)	0.481
Remifentanil	16 (39.2%)	8 (38.1%)	8 (40.0%)	0.574
Morphine	18 (43.9%)	9 (42.9%)	9 (45.0%)	0.567
Target temperature	Total (*n* = 41)	33 °C (*n* = 34)	36 °C (*n* = 7)	
Good neurologic outcome	21 (51.2%)	19 (55.9%)	2 (28.6%)	0.238

Values are expressed as median (interquartile ranges) or *n* (%) as appropriate.

*CPR* cardiopulmonary resuscitation, *ROSC* return of spontaneous circulation, *EEG* electroencephalography.

### Visual analysis of extracted EEGs

**Figure 1:**
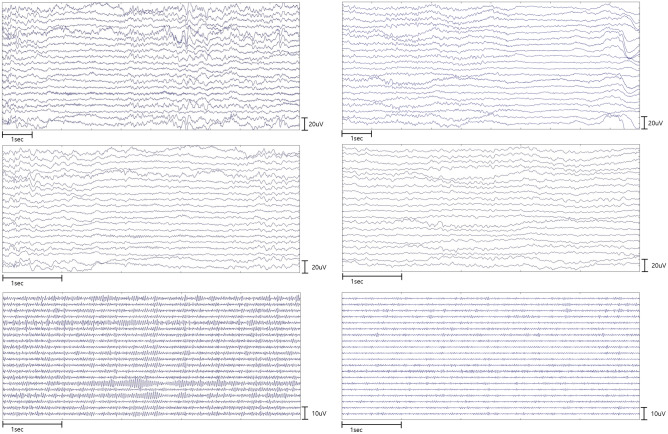
Representative visual analysis of raw data. The visual analysis could not differentiate poor and good neurological outcome. Example of EEGs from patients with poor (left panel) and good (right panel) neurological outcomes. Top—EEG filtered with a 0.5-Hz high-pass filter with a sensitivity of 10 μV/mm. Middle—EEG filtered with a 14-Hz high-pass filter with a sensitivity of 5 μV/mm. Bottom—EEG filtered with a 30-Hz high-pass filter with a sensitivity of 5 μV/mm. The alpha-PSD values of this epoch in patients with poor (left panel) and good (right panel) neurologic outcomes were 0.06 and 3.07, respectively.

Figure 1 shows examples of visual EEG analysis. Both patients in the good (*right*) and poor (*left*) neurologic outcome group showed symmetric, attenuated (all activities < 20 μV, 100% of record) background activities over whole record. There was no stage-II sleep transients or periodic rhythm. The dominant frequency and amplitude of background activities were difficult to differentiate between both patients in each group by visual analysis. In fact, the alpha-PSD values of this epoch in patients with poor (*left panel*) and good (*right panel*) neurologic outcomes were 0.06 and 3.07, respectively.

### Quantitative analysis of extracted EEGs according to background frequency

**Figure 2:**
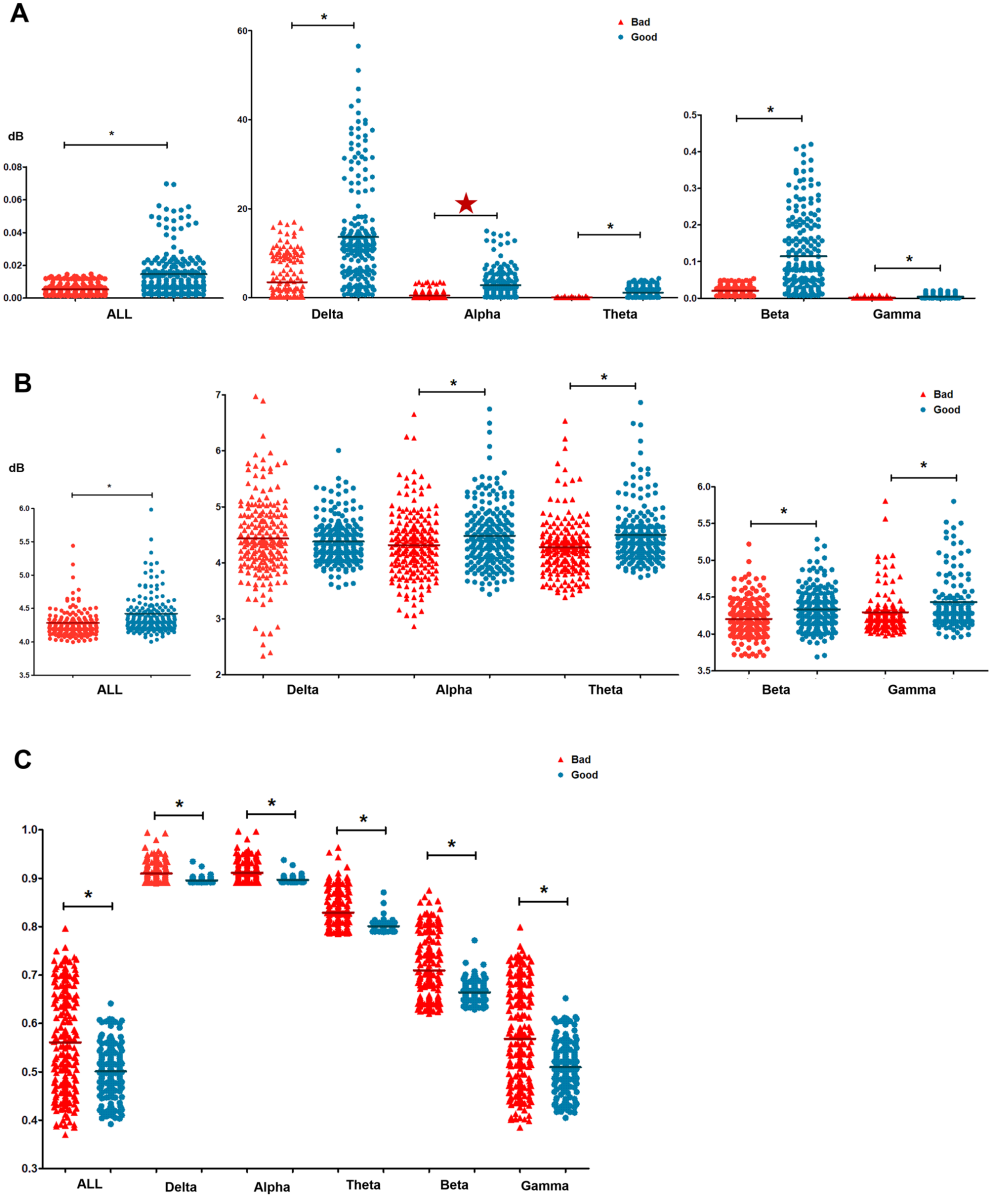
Scattered dots of (**A**) PSD, (**B**) ERSP, and (**C**) SEs of all and each frequency. Small star, *p* < 0.05; Large start, age-adjusted p < 0.05 by linear mixed model.

PSD, ERSP, and SE were compared between the patients in the good and poor neurologic outcome groups to quantify the power, variability, and randomness of each frequency band (Table 2; Fig. 2). All and each frequency band-PSD of patients with good neurologic outcomes were significantly higher compared to that with poor neurologic outcomes (Table 2; Fig. 2A). Except for the delta frequency band, all and the rest frequency band-ERSP of the patients in the poor neurologic outcome group were lower than the patients in the good neurologic outcome group with statistical significance (Table 2; Fig. 2B). Moreover, all and each frequency band-SE of the patients with poor neurologic outcomes were significantly higher than patients with good neurologic outcomes (Table 2; Fig. 2C). Only alpha-PSD was significantly higher in patients with good neurologic outcomes than those with poor neurologic outcomes after adjusting the age of each patient (Table 2; Fig. 2A).

**Table 2:** Comparisons of quantitative analysis using PSD, ERSP, and SE of each frequency between the out-of-hospital cardiac arrest patients with good and poor neurologic outcomes at 1 month.

Quantitative analysis according to each frequency		Good neurologic outcome (*n* = 21)	Poor neurologic outcome (*n* = 20)	*p* value	Age-adjusted *p* value
PSD	All	0.01 ± 0.01	0.01 ± 0.00	0.002	0.900
	Delta	13.65 ± 11.39	3.48 ± 4.64	< 0.001	0.837
	Theta	2. 79 ± 3.04	0.45 ± 0.76	0.002	0.793
	Alpha	1.13 ± 1.11	0.09 ± 0.09	< 0.001	0.031
	Beta	0.11 ± 0.10	0.02 ± 0.01	< 0.001	0.072
	Gamma	0.00 ± 0.00	0.00 ± 0.00	0.038	0.650
ERSP	All	4.42 ± 0.38	4.29 ± 0.31	0.004	0.295
	Delta	4.38 ± 0.39	4.43 ± 0.71	0.663	0.910
	Theta	4.48 ± 0.56	4.31 ± 0.57	0.046	0.091
	Alpha	4.45 ± 0.49	4.28 ± 0.49	0.028	0.653
	Beta	4.33 ± 0.26	4.21 ± 0.24	0.001	0.091
	Gamma	4.43 ± 0.54	4.29 ± 0.43	0.032	0.439
SE	All	0.50 ± 0.05	0.56 ± 0.11	0.014	0.092
	Delta	0.90 ± 0.00	0.91 ± 0.02	0.002	0.092
	Theta	0.90 ± 0.00	0.91 ± 0.02	0.002	0.095
	Alpha	0.80 ± 0.01	0.83 ± 0.04	0.002	0.071
	Beta	0.66 ± 0.02	0.71 ± 0.07	0.003	0.063
	Gamma	0.51 ± 0.05	0.57 ± 0.11	0.014	0.071

All frequency, 0.5–100 Hz; delta frequency, 0.5–4 Hz; theta frequency, 5–7 Hz; alpha frequency, 8–14 Hz; beta frequency, 15–30 Hz; and gamma frequency, 31–100 Hz.

*PSD* power spectral density, *ERSP* event-related spectral perturbation, *SE* spectral entropy.

### Useful biomarkers for predicting neurologic outcomes

**Figure 3:**
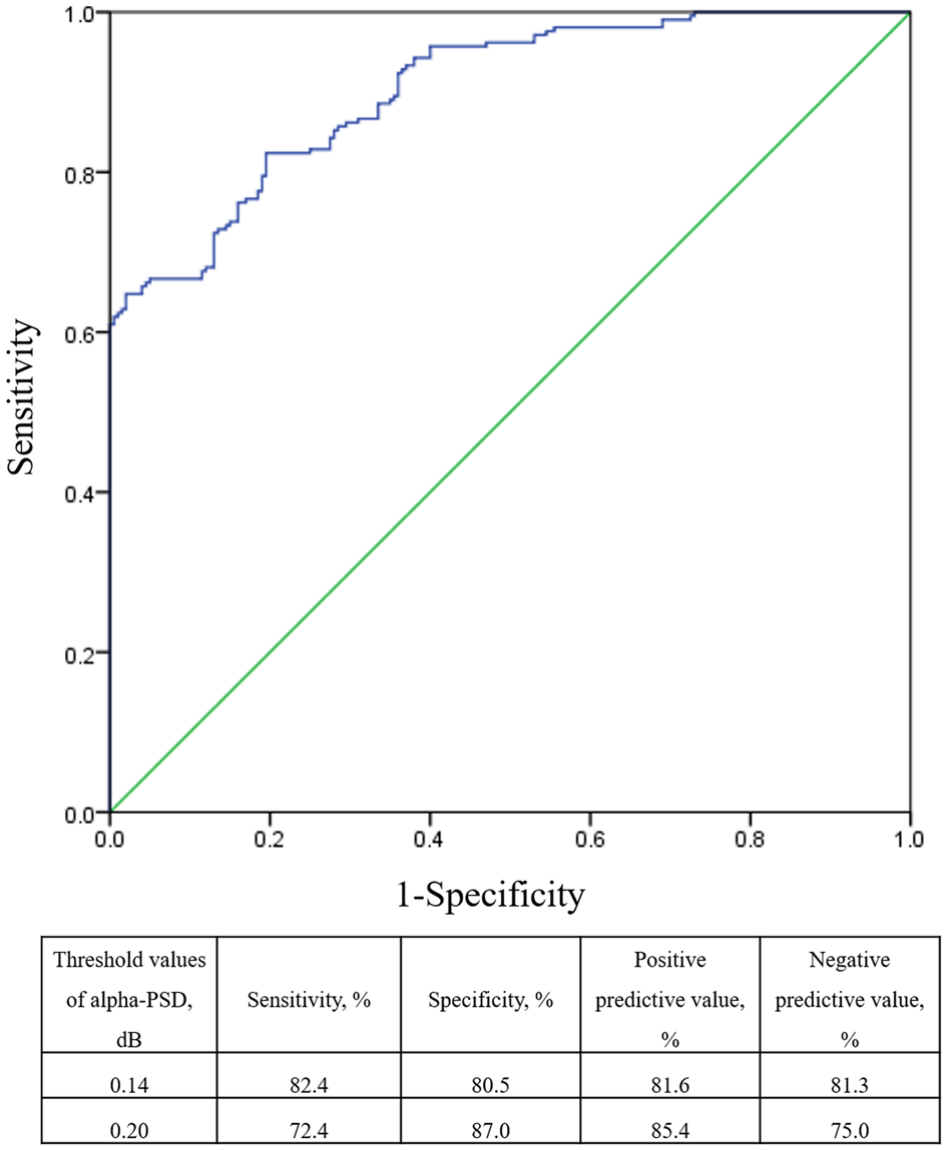
ROC curve (blue line) and predictive values according to the cutoff of alpha-PSDs; under the curve (AUC) of 0.903 and *p* value < 0.001.

Among three values of all and each frequency band, the alpha-PSD was the most reliable biomarker to differentiate the neurologic outcomes with the area under the curve (AUC) of 0.903 and *p* value < 0.001. The alpha-PSD cutoff values of the averaged C3 and C4 electrodes were 0.14 with 82.4% sensitivity and 80.5% specificity or 0.20 with 72.4% sensitivity and 87.0% specificity, respectively (Fig. 3).

## Discussion

In this study, we quantified the power, variability, and randomness of each frequency band in OHCA survivors with routine EEG to investigate the factor that can differentiate the good and poor neurological outcomes. We identify that the alpha-PSD was most reliable biomarker to indicate good neurologic outcomes with an AUC of 0.903.

Beyond the accurate description using Standardized Critical Care EEG Terminology, appropriate interpretation of these descriptions is essential for precise prognostication of neurologic outcomes in patients with OHCA. Among various background patterns defined in the Standardized Critical Care EEG Terminology, we found the dominant background frequency with alpha and theta waves were a powerful predictors (adjusted OR for favorable neurologic outcomes of 13.030) with high sensitivity (86.21%) and negative predictive value (91.30%) in our previous study^20^. However, the interrater variability is a challenging issue for neurologists and emergency physicians to accurately predict neurologic outcome and the agreement strength is decreased especially for background frequency interpretation^21^. In this study, the visual analysis of the EEG was also difficult to show the differences of predominant frequency between patients with poor and good neurologic outcomes even with time expansion with bandpass filter (Fig. 1). Thus, we focus the quantitative analysis to identify the power and dynamic changes of background brain oscillation according to each frequency band.

The brain has continuous neuronal oscillations even in resting-state and time-related variabilities and can reflect the subclinical brain dynamics during consciousness or unconsciousness. The ERSP has a strong ability to detect the time-related shift of the spectral powers from the baseline in a specific frequency^22–25^. Further, this analysis allows extracting information about the time course of power changes in each frequency under specific tasks including auditory stimuli, attention, and cognitive processes^26,27^ and hyperactive state of the brain with seizure or epileptic spasms in previous studies^28,29^.

Under steady-state condition, the neuronal firing is constantly occurred to transfer the information between neuron, and a synchronous state is maintained. The weakly correlated action potentials of individual neurons, for any reason, can lead to the asynchronous state and increase randomness^30–32^, entropy. The entropy algorithms have already been widely used in analyzing depth of anesthesia^33,34^ and detecting seizures^35,36^.

To identify the time-related variability and randomness of power in each frequency band, we calculate the ERSP and SE in this study, respectively. No significant differences of ERSP and SE in all frequencies between the groups after age adjustment indicated that the patients with OHCA had too low brain activities to measure the variability or randomness without external stimulation due to acute neuronal damage itself or treated sedatives.

The PSD analysis is a method for quantitatively evaluating the power distribution of target frequency ranges. The low amplitude of background activity of < 20 μV in the entire channels, including suppressed or attenuated EEG pattern, is classified as highly malignant or malignant EEG that predicts a poor neurologic outcome^12^. In these context, we quantify the background power of each frequency using PSD and identify that the powers of alpha frequency bands are increased in the good neurologic outcome group in line with previous studies^12,37^.

Regarding dominant background frequency, we are interested in alpha frequency band, which is related to the good neurological outcome in our previous and current studies^20^. Alpha oscillations are the salient features during resting or wakefulness, and the change of alpha frequency is related to aging, cognitive function, sensorimotor processing, sleep, and neurodegenerative disease including Alzheimer’s disease^38–40^. A recent study with OHCA patients reported that the unfavorable outcome group showed lower power spectra in around 10 Hz alpha frequency compared with the favorable outcome group^41^. Furthermore, another study found that the prominent alpha peaks were presented in patients with minimally conscious states and in none of the comatose patients^42^. Consistently, the power of alpha frequency is higher in the good outcome group than in the poor outcome group after adjustment by age and the most reliable biomarker to predict neurologic outcomes in patients with OHCA.

To our knowledge, this is first report that quantify the three characteristics of the dominant background frequency to predict neurologic outcomes in OHCA patients. The findings of the current study suggest that alpha-PSD can be used as simple and reliable biomarkers for prognostication of good neurologic outcomes. There were only few studies to predict the good neurologic outcome for OHCA patients and alpha-PSD could be one of the valuable and accurate biomarkers to predict good neurologic outcome^20,43,44^. The results of the current study should be interpreted in the context of the following limitations. The retrospective study design and associated selection bias may have influenced the results. Moreover, the limited number of patients may have contributed to the lack of significance of some results of the current study. Another potential concern is the EEG timing and influence of sedative agents during EEG recording. Additionally, we used the data of patients with continuous background, who potentially had less severe spectrum of anoxic injury. However, the use of sedative agents followed the postcardiac arrest care protocol in this experienced tertiary center, and a relative homogenous standard routine EEG protocol was used. In addition, the cohort of the current study is not complicated by the WLST issue due to forbidden by law in South Korea during the study period. This results mainly focused EEG biomarker and delicate applications with other prognostic factors in actual clinical field should be considered according to present guidelines^45^.

Considering the poor interrater agreement and reproducibility of the visual interpretation of standard intermittent EEG, the quantitative analysis method with PSD, ERSP, and SE feature extraction presented in this study suggests alpha-PSD as early prognostic tools for neurologic outcome in patients with OHCA. Further larger studies are warranted to validate the predictive value of the alpha-PSD of early standard intermittent EEG proposed in this study.

## Methods

### Study design and patients

This retrospective, observational, registry-based cohort study was conducted at the emergency intensive care unit (ICU) of a tertiary care university-affiliated teaching hospital in Korea. Data were extracted from the OHCA registry containing prospectively collected data of consecutive patients with OHCA since January 2010^46^. The Institutional Review Board of the University of Ulsan College of Medicine, Seoul, Korea, reviewed and approved the study protocol (no. 2019-1883). Informed consent was waived due to the retrospective nature of the study by Institutional Review Board of the University of Ulsan College of Medicine, Seoul, Korea (no. 2019-1883). All experiments were performed in accordance with relevant guidelines and regulations.

This study included patients with successfully resuscitated nontraumatic OHCA > 18 years old and treated with TTM due to neurologic impairments after the return of spontaneous circulation (ROSC) between January 2014 and December 2018. Routine EEG was recorded in the first 48 h after ROSC. All patients were comatose during EEG recording and TTM. We excluded patients with poor EEG quality which is impossible to extract the representative 5-min EEG or periodic/rhythmic EEG pattern. The excluded periodic or rhythmic pattern were represented in supplementary Fig. 1. All patients were followed up to 1 month after cardiac arrest with neurologic assessment using the Cerebral Performance Category (CPC) score. The primary outcome of this study was a good neurologic outcome at 1 month, defined as CPC scores 1 and 2.

### Management and data collection

All patients were treated according to the current advanced cardiac life support guidelines^47,48^. TTM was performed for all unconscious patients using Arctic Sun Energy Transfer Pad (Medivance Corp., Louisville, CO, USA), and the target temperature (33 °C or 36 °C) was maintained for 24 h. Patients were rewarmed at a rate of 0.25 °C/h after 24 h, following maintained normothermia until 72 h from ROSC. The temperature was monitored using an esophageal temperature probe. A combination of propofol, midazolam, fentanyl, remifentanil, and morphine was used for sedation and analgesia. A neuromuscular blocking agent was administered to control shivering if necessary. Patients with seizure activity on EEG were treated with valproate or levetiracetam. All patients received standard intensive care according to institutional protocol. WLST was legally prohibited in South Korea until August 2017^49^, and none of the patients in the current study underwent WLST.

Demographic and clinical data, including age, sex, previous medical history, resuscitation profiles such as the presence of a witness on collapse, initial documented rhythm, and resuscitation duration) were obtained.

### EEG recording and preprocessing

Standard EEG examination was started during TTM after ICU admission. The 30-min scalp EEG was recorded by the Stellate EEG system from 21 electrodes that were placed according to the international 10–20 electrode system (Fp1–2, F7–8, T7–8, P7–8, F3–4, C3–4, P3–4, O1–2, Fz, Cz, and Pz) with a sampling rate of 200 and 0.1 Hz high-pass filter. EEGs were initially reviewed to select clean data without apparent artifacts or muscle activities, and 5 min of each artifact-free resting-state EEG were collected. The extracted EEGs were extended temporally, and 0.5, 15, and 30 Hz high-pass filters were used to differentiate visible background amplitude and frequency, including beta (14–30 Hz) and gamma (30–100 Hz) with sensitivities of 10 and 5 μV/mm, respectively (Fig. 1). Before quantitative analysis, the data were preprocessed using the EEGLAB toolbox of MATLAB 2020a. The 0.5-Hz high-pass and 60-Hz notch filters were used to remove the alternating current at 60 Hz, and independent component analysis was applied using *runica (),* plugged in EEGLAB, to remove artifacts. Finally, the extracted 5 min of EEG data of each patient were divided into 10 epochs (30-s duration) per patient. The EEG from the C3 and C4 electrodes were selected for data extraction to reduce the muscle and eyeball movement artifacts.

### Spectral power analysis (power spectral density)

The PSD estimated the power of each frequency band directly from the signal itself. The PSD (log) was computed by Welch power spectral estimation for each patient with a sampling rate (200 Hz) and 15-s window size length segments in each 30-s epoch. The average in each PSD frequency including delta (0.5–4 Hz), theta (5–7 Hz), alpha (8–14 Hz), beta (15–29 Hz), and gamma (30–100 Hz) extracted from the C3 and C4 electrodes of 10 epochs were calculated.

### Variability analysis (event-related spectral perturbation)

ERSP (log) was used to show dynamic brain changes with the zero point in each epoch set as the baseline. ERSPs were analyzed with fast Fourier transform and Hanning window tapering in each 30-s epoch. The mean baseline log power spectrum was subtracted to produce the baseline-normalized ERSP from each spectral estimate, and then deviations from baseline power were calculated. The detail method was described in supplementary method. The averaged each frequency ERSP including delta (0.5–4 Hz), theta (5–7 Hz), alpha (8–14 Hz), beta (15–29 Hz), and gamma (30–100 Hz) extracted from the C3 and C4 electrodes of 10 epochs were calculated.

### Randomness analysis (spectral entropy)

SE based on Shannon entropy in physics, quantifying the regularity/randomness of power spectrum during a given time, was used to establish one of the biomarkers for neurological prognosis after cardiac arrest in the recent study^50^. In effect, SE reflected the randomness of the power spectrum distribution. The greater SE represents the more uniform power spectral distribution^51^. The detail method was described in supplementary method. The average of each frequency SE including delta (0.5–4 Hz), theta (5–7 Hz), alpha (8–14 Hz), beta (15–29 Hz), and gamma (30–100 Hz) were extracted from C3 the C4 electrodes of 10 epochs were calculated.

### Statistical analysis

Continuous variables are presented as median with interquartile range (IQR) due to the non-normal distribution using the Kolmogorov–Smirnov test. In addition, categorical variables are expressed as absolute numbers and percentages. The patients were categorized into two groups based on their CPC scores at 1 month: good neurologic (CPC 1 and 2) and poor neurologic (CPC 3–5)^52^ neurologic outcome groups were performed using the Mann–Whitney *U*-test and chi-square test for continuous and categorical variables, respectively.

The linear mixed model analysis for PSD, ESRP, and SE values for all and each frequency band was performed to adjust the random effect of clustering from the epochs of the same patients at 10 time-points and fixed effect of ages of each patient. The age could have an influence on EEG rhythmic activities including delta, theta, alpha, beta, and gamma^52^ and we adjusted the ages as fixed effect in linear mixed model analysis. Each variable was compared between the good and poor neurologic outcome groups, and receiver operating characteristic curves with 95% confidence intervals for good neurologic outcomes were analyzed to determine the cutoff values to predict neurologic prognosis. Sensitivity and specificity for good neurological outcomes at 1 months were calculated. All statistical analyses were performed using IBM SPSS for Windows, version 21.0 (IBM Corp., Armonk, NY, USA).

## Supplementary Information

Supplementary Information 1.

Supplementary Information 2.

## Data Availability

The datasets used and/or analysed during the current study available from the corresponding author on reasonable request.
