# Supplementary material for: Alpha-power in electroencephalography as good outcome predictor for out-of-hospital cardiac arrest survivors
Source: Sci Rep. 2022 Jun 28;12:10907. doi: 10.1038/s41598-022-15144-3 (PMC9240023; doi:10.1038/s41598-022-15144-3)
Supplement: Supplementary file 1 — Supplementary Information 1. [file 41598_2022_15144_MOESM1_ESM.docx]

**Supplementary methods**

**Variability analysis (event-related spectral perturbation)**

The ERSP formula for averaged estimates across data trials (*n* trials) is defined below:

ERSP (*f*, *t*) $=\frac{1}{n}\sum_{k=1}^{n} \left| F_{k}\left( f,t \right) \right|^{2}$

where $F_{k}\left( f,t \right)$ is the spectral estimate of trial *k* at frequency *f* and time *t*.

**Randomness analysis (spectral entropy)**

Using previously calculated PSD (*P_sd(f)_*) via Welch’s method, the normalized PSD, which was defined as the *P_sd(f)_* divided by the total power of each frequency band, was analyzed to obtain probability density function.

$$\overline{Psd\left( f \right)}= \frac{Psd(f)}{\sum_{f=a}^{f=b} Psd(f)}$$

where $\overline{Psd\left( f \right)}$ was the normalized PSD of *P_sd(f)_*. SE was estimated based on the PSD within each frequency band: *a* = 0.5 and *b* = 4 for delta-$\overline{Psd\left( f \right)}$; *a* = 5 and *b* = 7 for theta-$\overline{Psd\left( f \right)}$; *a* = 8 and *b* = 14 for alpha-$\overline{Psd\left( f \right)}$; *a* = 15 and *b* = 29 for beta-$\overline{Psd\left( f \right)}$, and *a* = 30 and *b* = 100 gamma-$\overline{Psd\left( f \right)}$. The SE ($SEn$) was generated by using the following equation:

$$SEn= -k\sum_{f=a}^{f=b} \overline{Psd\left( f \right)}log(\overline{Psd\left( f \right)})$$

where *k =* 1.

**Supplementary figure 1.** Excluded periodic or rhythmic pattern in this study.
