# Supplementary figures and images for: Alpha-power in electroencephalography as good outcome predictor for out-of-hospital cardiac arrest survivors
Source: Sci Rep. 2022 Jun 28;12:10907. doi: 10.1038/s41598-022-15144-3 (PMC9240023; doi:10.1038/s41598-022-15144-3)

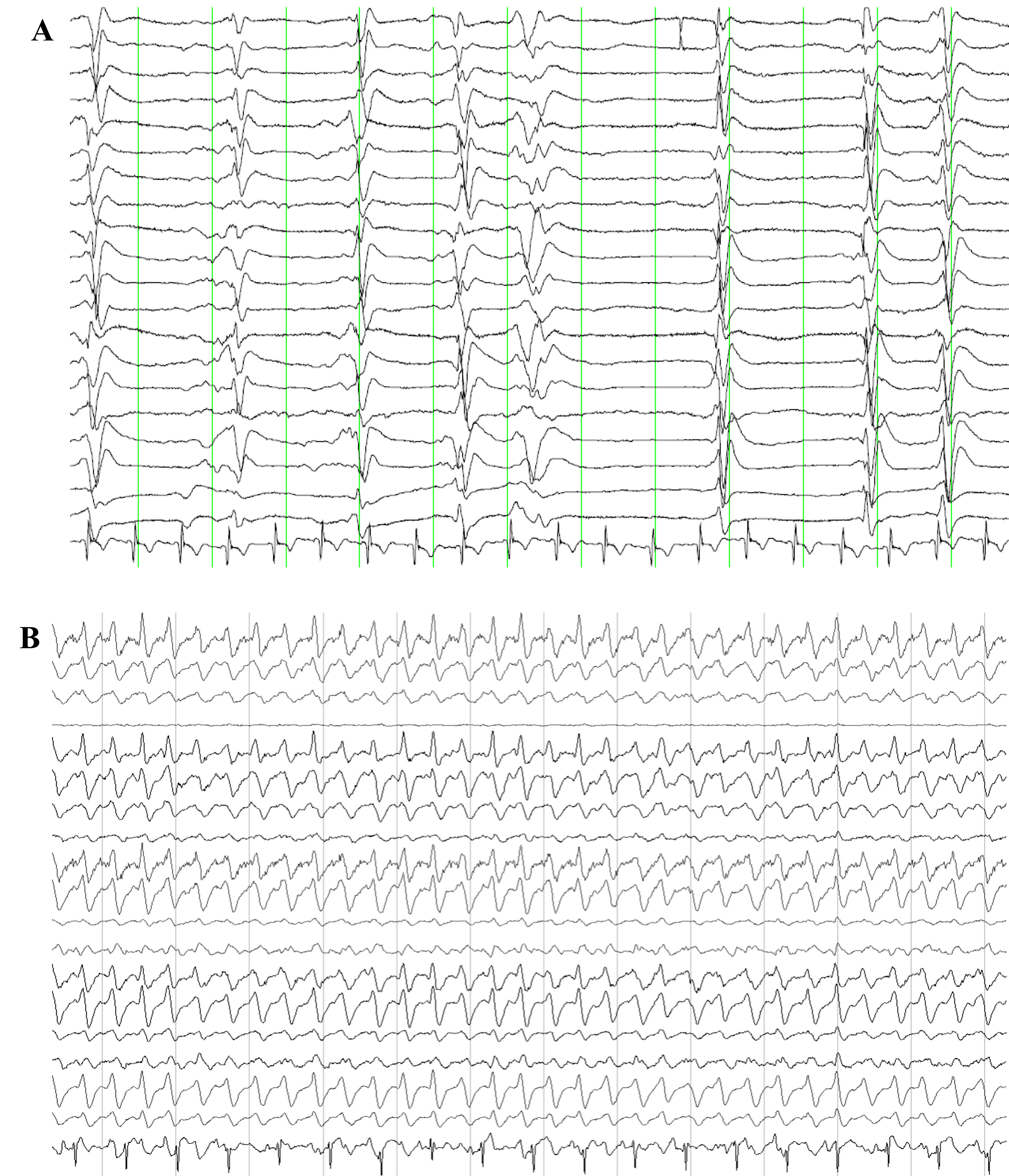

Supplement: Supplementary file 2 — Supplementary Information 2. [file 41598_2022_15144_MOESM2_ESM.tif]
